# Two-dimensional ballistic transistors for advanced-node integrated circuits

**DOI:** 10.1093/nsr/nwad315

**Published:** 2023-12-13

**Authors:** Jia Li, Xidong Duan

**Affiliations:** Hunan Provincial Key Laboratory of Two-Dimensional Materials, State Key Laboratory for Chemo/Biosensing and Chemometrics, College of Chemistry and Chemical Engineering, Hunan University, China; Hunan Provincial Key Laboratory of Two-Dimensional Materials, State Key Laboratory for Chemo/Biosensing and Chemometrics, College of Chemistry and Chemical Engineering, Hunan University, China

As the feature size of metal-oxide-semiconductor-field-effect-transistors (MOSFETs) in integrated circuits (ICs) is reduced to sub-10-nm, the traditional channel materials are plagued by enhanced scattering and short-channel effects, leading to the deterioration of switching characteristics and the increase of power consumption. Two-dimensional atomic crystals (2DACs) with atom-scale thickness and dangling bond-free surfaces have shown great potential as promising channel materials for extreme device scaling. According to the 2021 *International Roadmap for Devices and Systems* (IRDS), 2DACs could potentially be a strong competitor for silicon FETs at the sub–1.0-nm node [1].

To explore the ultimate potential of 2DAC FETs, there are three prerequisites: (i) producing high-quality 2D semiconductors with a small effective mass and a small scale length; (ii) constructing ultra-thin high-k dielectrics and high-quality gate-stack interfaces to improve the electrostatic gate control; (iii) constructing high-quality semiconductor-metal (MS) ohmic contact to decrease contact resistance (*R*_C_) [2]. Many studies have shown that the actual device performance is mainly limited by the Fermi pinning effect (FPE) in non-ideal MS interfaces. Various strategies, including van der Waals integration of 2DACs and metals [3,4], semi-metal Bi or Sb contacts [5,6], low energy deposition of 3D metals [7], and phase transition [8] have been developed to alleviate FPE. Although *R*_C_ obtained some significant improvement, the overall performance of 2D FETs is still far from the theoretical prediction, lagging far behind that of state-of-the-art silicon MOSFETs.

Recently, writing in *Nature*, J. Jiang and colleagues have reported the first realization of 2D ballistic transistors with n-type indium selenide (InSe) as channel material (Fig. 1a) [9]. To obtain ohmic contact in InSe FETs, the key is introducing a semi-metal yttrium-doping InSe as the buffer layer between the MS interface by a phase-transition process (Fig. 1b). Notably, the InSe FET has a constant on-state current (1 mA·μm^−1^) over a wide temperature range (300–100 K), providing strong evidence for the achievement of ballistic transistors with ohmic contacts (Fig. 1c). As-fabricated InSe FETs with a 10-nm channel length and double-gate configuration (2.6-nm–thick HfO_2_ dielectrics) can effectively suppress short-channel effects and show nearly perfect switching behaviour with a low supply voltage of 0.5 V, a record high transconductance 6 mS·μm^−1^ and room-temperature ballistic ratio in the saturation region of 83% (Fig. 1c and d). Furthermore, a low *R*_C_ of 62 Ω·μm was reliably extracted in 10-nm ballistic InSe FETs, leading to a faster speed and much lower energy consumption than the predicted silicon limit.

**Figure 1.**
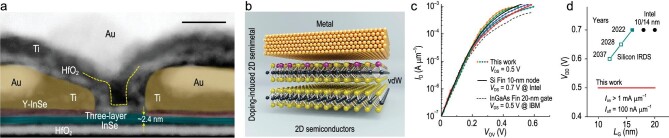
Two-dimensional ballistic InSe transistors. (a) Transmission electron microscopy image showing a cross-section of an InSe FET with a double-gate structure. (b) Schematic of a semi-metal Y-doping contacted InSe FET. (c) Transfer characteristics comparison of five typical ballistic 2D InSe FETs (coloured dots), 10-nm-node silicon FinFET (Intel, solid black line), and 20-nm gate InGaAs FinFET normalized by state-of-the-art Fin Pitch = 34 nm (IBM, dashed black line). (d) Scaling trends of *V*_DD_ of the ballistic 2D InSe FETs compared with those of silicon FETs (the silicon data are from IRDS 2022 and Intel). (a, c and d) Reprinted with permission from [9]. (b) Reprinted with permission from [12].

This work [9] for the first time fabricates 2D ballistic transistors with whole performance surpassing those of state-of-the-art silicon FETs. However, this work relies on the mechanically exfoliated InSe with small sizes, large-scale integration of such devices for ICs needs the production of wafer-scale high-quality InSe single crystals, which require more effort. The stability of semi-metal Y-doping contacts for heat/wet resistance also needs further exploration. Encouragingly, this work, together with several representative works published recently, such as 2D FETs with ultra-high on-state current density (1.72 mA/μm for bilayer WSe_2_) [10], 2D ICs operating at gigahertz frequencies [11], strongly demonstrate the superiority, feasibility and enormous potential of 2DACs in the post-Moore electronics.

## References

[1] IRDS . IEEE International Roadmap for Devices and Systems (IRDS, 2021). https://irds.ieee.org/editions/2021 (11 December 2023, date last accessed).

[2] Liu Y, Duan X, Shin HJ et al. Nature 2021; 591: 43–53. 33658691 10.1038/s41586-021-03339-z

[3] Liu Y, Guo J, Zhu E et al. Nature 2018; 557: 696–700. 29769729 10.1038/s41586-018-0129-8

[4] Li J, Yang X, Liu Y et al. Nature 2020; 579: 368–74. 32188941 10.1038/s41586-020-2098-y

[5] Shen PC, Su C, Lin Y et al. Nature 2021; 593: 211–7. 33981050 10.1038/s41586-021-03472-9

[6] Li W, Gong X, Yu Z et al. Nature 2023; 613: 274–9. 36631650 10.1038/s41586-022-05431-4

[7] Wang Y, Kim JC, Li Y et al. Nature 2022; 610: 61–6. 35914677 10.1038/s41586-022-05134-w

[8] Cho S, Kim S, Kim JH et al. Science 2015; 349: 625–8. 26250680 10.1126/science.aab3175

[9] Jiang J, Xu L, Qiu C et al. Nature 2023; 616: 470–5. 36949203 10.1038/s41586-023-05819-w

[10] Wu R, Tao Q, Li J et al. Nat Electron 2022; 5: 497–504.

[11] Fan D, Li W, Qiu H et al. Nat Electron 2023; 6: 879–87.

[12] Jiang J, Xu L, Du L et al. Res Square 2023; doi: 10.21203/rs.3.rs-2508636/v1 10.21203/rs.3.rs-2508636/v1.

